# Cutaneous Herpes Zoster Infection as a Rare Etiology of Syndrome of Inappropriate Antidiuretic Hormone Secretion (SIADH): A Case Report

**DOI:** 10.7759/cureus.69446

**Published:** 2024-09-15

**Authors:** Andrea Quartey, Monica Patel

**Affiliations:** 1 Internal Medicine, Temple University Hospital, Lewis Katz School of Medicine at Temple University, Philadelphia, USA

**Keywords:** herpes zoster virus, immunosuppression, shingles complications, siadh, varicella zoster virus

## Abstract

Herpes zoster is caused by a reactivation of the varicella-zoster virus after primary manifestation during childhood as chicken pox, commonly occurring in elderly and immunosuppressed patients. Syndrome of Inappropriate Antidiuretic Hormone Secretion (SIADH) is defined by the unsuppressed release of antidiuretic hormone (ADH) from the pituitary gland, with impaired water excretion causing hyponatremia. Though limited, literature supports herpes zoster infection inducing SIADH, through a proposed mechanism of alteration of the ADH regulator pathway due to viral infection of the dorsal root ganglion (DRG). Our case is distinct in that our patient presented elements of hypovolemic hyponatremia with concomitant SIADH.

## Introduction

Herpes zoster (HZ) is caused by human herpesvirus, varicella zoster virus (VZV), and manifests commonly in the elderly or immunosuppressed. This is due to the reactivation of the virus after primary infection (colloquially known as chicken pox) in childhood [1]. Syndrome of Inappropriate Antidiuretic Hormone Secretion (SIADH) is a condition defined by the unsuppressed release of antidiuretic hormone (ADH) from the pituitary gland or non-pituitary sources, as well as the hormone’s continued action on vasopressin receptors [2]. It is characterized by impaired water excretion leading to euvolemic hyponatremia or hypervolemic hyponatremia [2]. We propose a distinct clinical presentation in this patient who presented with severe hyponatremia while acutely ill with HZ; specifically, hypovolemic hyponatremia with concomitant SIADH in the setting of HZ infection.

## Case presentation

**Figure 1:**
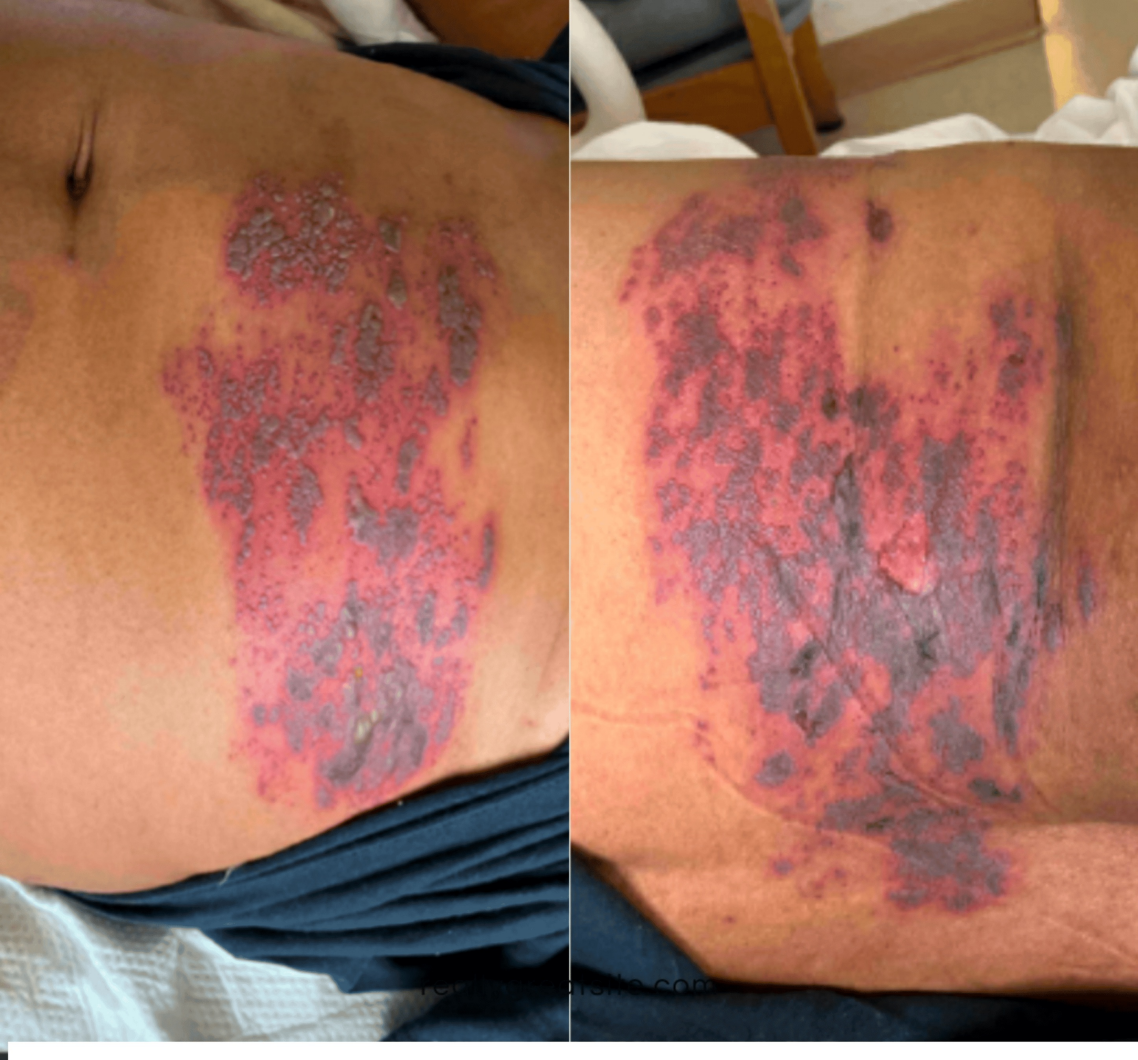
Vesicular, blistering, and violaceous rash in various stages of healing on anterior and posterior T6-T7 trunk.

A 65-year-old man presented with one week of nausea, vomiting, and diarrhea (N/V/D), along with poor oral intake. His medical history was notable for a diagnosis of VZV infection managed in the outpatient setting, as well as marginal zone lymphoma, for which he had been undergoing eight months of treatment with R-CHOP (rituximab, cyclophosphamide, hydroxydaunorubicin hydrochloride, vincristine, and prednisone) chemotherapy. The patient completed his fifth and most recent cycle of chemotherapy three months prior to the current admission. Notably, he was not currently receiving corticosteroids. Ten days prior to admission, the patient developed HZ, and outpatient treatment with acyclovir was initiated seven days prior to admission. Of note, the patient had not received the shingles vaccine. He was febrile, with a temperature around 38.4^o^C on presentation but was otherwise hemodynamically stable. Physical examination revealed a violaceous, vesicular rash spanning T6-T7 dermatomes along the left anterior and posterior trunk (Figure 1). On laboratory examination, the patient was severely hyponatremic, with a sodium level of 119 mg/dL. Patient labs reported that thyroid, adrenal, and kidney function were within normal limits.

The patient, who was in an immunocompromised state, underwent a comprehensive infectious workup after presenting with a fever. The workup, including contrast-enhanced tomography of the chest, abdomen, and pelvis, as well as urinalysis, blood cultures, viral etiologies, and stool studies were all negative for acute infections. The patient's fever resolved without the need for targeted antibiotic therapy and was attributed to an active VZV infection, for which he had been previously treated with acyclovir in outpatient care. The patient was restarted on acyclovir 800 mg five times daily due to uncertainty regarding the extent of drug absorption, given the significant gastrointestinal losses from N/V/D. The medication was tolerated well throughout his hospital stay. In addition to the fever, he was noted to be hyponatremic with serum sodium at 119 mg/dL on admission. Urine studies were performed to better characterize the type of hyponatremia and were indicative of hypo-osmolar hyponatremia (Table 1).

**Table 1:** Patient hyponatremia studies consistent with SIADH Comparison of the patient's laboratory values associated with hyponatremia revealing SIADH versus normal reference ranges. The table highlights key electrolyte imbalances and relevant clinical parameters that are indicative of SIADH, including serum sodium, serum osmolarity, urine osmolarity, and urine sodium levels. SIADH: Syndrome of Inappropriate Antidiuretic Hormone Secretion; mEq/L: milliequivalents per liter; mOsm/kg: miliosmoles per kilogram of water

	Patient Laboratory Values	Normal Reference Ranges
Serum Sodium	119 mEq/L	135-145 mEq/L
Serum Osmolality	256 mOsm/kg	275-295 mOsm/kg
Urine Osmolality	422 mOsm/kg	50-1200 mOsm/kg
Urine Sodium	32 mEq/L	20-40 mEq/L

Due to initial concerns about hypovolemia, attributed to the patient's N/V/D, as well as his inability to tolerate oral intake on admission, the patient underwent aggressive fluid resuscitation with over 5 liters of normal saline over the course of 24 hours. However, there was minimal improvement in sodium levels. After adequate volume resuscitation, the patient was placed on fluid restriction, as urine studies were consistent with SIADH, with urine osmolality > 100. His sodium level improved and was sustained at 130 mg/dL after fluid restriction until the day of discharge.

## Discussion

Hyponatremia is a common electrolyte abnormality in hospitalized patients and may stem from several etiologies. In this patient, the hyponatremia was determined to be multifactorial, with contributions from both hypovolemic and SIADH-related mechanisms. Early in the clinical course, the patient exhibited signs of hypovolemia, likely related to N/V/D. Despite fluid resuscitation, the hyponatremia persisted, suggesting that factors beyond volume depletion were involved. 

SIADH was suspected of being a significant contributor based on further investigation. Hyponatremia secondary to SIADH is commonly associated with conditions such as lung disease, drugs like selective serotonin reuptake inhibitors (SSRIs), and central nervous system (CNS) disorders [3]. Any CNS disturbance can precipitate the enhanced ADH secretion from the pituitary gland, precipitating SIADH [2]. VZV infection is classified as a CNS disease due to its latency in the dorsal spinal cord, stemming from childhood varicella chickenpox. The incidence of VZV is increased in patients with decreased immune function such as the elderly and those undergoing chemotherapy [1], both of which apply to the current patient. While SIADH in the setting of VZV is rare, its potential manifestations should be noted for the future management of patients. The patient displayed hyponatremia deemed to be multifactorial in nature upon discharge. There appeared to be an element of hypovolemic hyponatremia (due to N/V/D but persisted after fluid administration) and SIADH due to VZV reactivation. VZV reactivation has presented with a noted effect on regulatory pathways of ADH secretion by previous case reports [4]. While SIADH in association with HZ is already distinct, what is especially unique in this patient presentation is that he exhibited multifactorial etiologies of hyponatremia, notably low sodium levels in the setting of VZV infection.

The specific mechanism of HZ-induced SIADH is vague within the literature; however, Wang et al. suggest that VZV latency in sensory neurons of dorsal root ganglia (DRG) plays a role. The DRG is composed of numerous neurons with different functions such as relaying messages from peripheral thermal, pain, pressure, vibration, and proprioception receptors. Upon viral reactivation from the DRG in old age and waning immunosuppression, VZV spreads to the corresponding dermatome via the axons of infected neurons. It then spreads to the dorsal columns of the spinal cord. Peripheral osmoreceptors are thought to travel partially through the DRG before reaching the spinal cord and CNS. From there, they reach the supraoptic and paraventricular nuclei of the hypothalamus, subsequently affecting the pituitary gland [4,5]. If VZV infects neurons responsible for peripheral osmoreceptors, it is plausible to suspect that this mechanism could affect the inputs and regulation of ADH secretion. Though SIADH in VZV reactivation is rare, it should be considered in similar cases, especially when hyponatremia persists despite volume correction.

Additionally, the potential for hypovolemic hyponatremia complicating this patient's presentation emphasizes the importance of considering multifactorial etiologies in hospitalized patients. As per the literature, there is a potential causal relationship between acyclovir treatment and the development of hyponatremia [6]. However, this situation is less likely in this patient’s case due to poor oral intake of medication prior to hospital admission, where there is uncertainty about the amount of true acyclovir intake.

## Conclusions

Our patient represents a case of hyponatremia that is multifactorial in etiology, in association to his active HZ infection. It demonstrates that it is imperative for clinicians to become increasingly aware of the possibility of hyponatremia stemming from several components when considering the underlying cause of patient hyponatremia. This helps to ensure swift and effective management of hyponatremic patients.
